# Unmixing Oscillatory Brain Activity by EEG Source Localization and Empirical Mode Decomposition

**DOI:** 10.1155/2019/5618303

**Published:** 2019-03-14

**Authors:** Sofie Therese Hansen, Apit Hemakom, Mads Gylling Safeldt, Lærke Karen Krohne, Kristoffer Hougaard Madsen, Hartwig R. Siebner, Danilo P. Mandic, Lars Kai Hansen

**Affiliations:** ^1^Cognitive Systems, Department of Applied Mathematics and Computer Science, Technical University of Denmark, Richard Petersens Plads B321, DK-2800 Kgs. Lyngby, Denmark; ^2^Department of Electrical and Electronic Engineering, Imperial College London, South Kensington Campus, Kensington, London SW7 2AZ, UK; ^3^Danish Research Centre for Magnetic Resonance, Centre for Functional and Diagnostic Imaging and Research, Copenhagen University Hospital Hvidovre, Kettegaard Allé 30, DK-2650 Hvidovre, Denmark; ^4^Department of Neurology, Copenhagen University Hospital Bispebjerg, Bispebjerg Bakke 23, DK-2400 Copenhagen NV, Denmark

## Abstract

Neuronal activity is composed of synchronous and asynchronous oscillatory activity at different frequencies. The neuronal oscillations occur at time scales well matched to the temporal resolution of electroencephalography (EEG); however, to derive meaning from the electrical brain activity as measured from the scalp, it is useful to decompose the EEG signal in space and time. In this study, we elaborate on the investigations into source-based signal decomposition of EEG. Using source localization, the electrical brain signal is spatially unmixed and the neuronal dynamics from a region of interest are analyzed using empirical mode decomposition (EMD), a technique aimed at detecting periodic signals. We demonstrate, first in simulations, that the EMD is more accurate when applied to the spatially unmixed signal compared to the scalp-level signal. Furthermore, on EEG data recorded simultaneously with transcranial magnetic stimulation (TMS) over the hand area of the primary motor cortex, we observe a link between the peak to peak amplitude of the motor-evoked potential (MEP) and the phase of the decomposed localized electrical activity before TMS onset. The results thus encourage combination of source localization and EMD in the pursuit of further insight into the mechanisms of the brain with respect to the phase and frequency of the electrical oscillations and their cortical origin.

## 1. Introduction

Neuronal oscillations occurring in synchrony or asynchrony are fundamental for cognitive processes, and their study is important for understanding the healthy and diseased brain [1–3]. Due to the time scale of these oscillations, they are best captured by electroencephalography (EEG) and magnetoencephalography (MEG). Temporal, spatial, and frequency content of the brain dynamics can be exploited in, e.g., making brain-computer interfaces (BCIs) [4, 5] and neurofeedback [6], and for optimizing brain stimulation [7], e.g., in decreasing interindividual and intraindividual variability of transcranial magnetic stimulation (TMS) [8–11]. In order to derive meaning from the brain's diverse oscillation patterns, it is helpful to decompose the measured signal in space and time.

The temporal dynamics of the neuronal oscillations are often characterized by their frequency, phase, and amplitude. The empirical mode decomposition (EMD) technique [12–14] facilitates a data-driven, adaptive extraction of distinct time series from which these characteristics can be derived. Instead of forming the decomposition of a signal on a predefined basis, like when using standard Fourier or wavelet approaches, the basis of, e.g., the brain oscillations is estimated from the signal itself. EMD thus has the advantage of being able to handle nonlinear and nonstationary signals, which are often encountered in EEG/MEG [15, 16]. The *instantaneous* frequency, phase, and amplitude of the different EEG/MEG components can therefore be determined. EMD has been employed in several applications, e.g., seismology [17] and BCIs [18].

While the temporal resolution of EEG is high, making time-frequency analysis of this type of brain signal ideal, its spatial resolution is quite low [19]. This is partly due to the low number of recording sensors compared to the very high number of neuronal generators. Further reduction in spatial resolution is caused by volume conduction, which produces a smearing of the brain signals as seen by the sensors. Unmixing the signal can partly be achieved by localizing the cortical origin of the measured EEG signal, i.e., through source localization. This corresponds to solving the inverse problem of EEG. As the number of sensors is small compared to the number of potential neuronal generators, this is an ill-posed problem. Assumptions on the source distribution must therefore be made, such as spatial/temporal smoothness and/or spatial sparsity [20–27]. Several studies have shown that source localization compared to scalp EEG increases the amount of information that can be extracted. Besserve et al., for example, showed that source localization improves decoding in BCIs [28], and Edelman et al. found that similar hand movements were more easily distinguished at the brain-source level than at the sensor level [29]. Furthermore, Andersen et al. observed that the perception of faces could be better separated from that of scrambled faces using source localization [30]. Signal decomposition of source-localized electrophysical activity has in general shown promising results. Luckhoo et al. recovered task relevant brain networks by applying independent component analysis in combination with the general linear model to the envelope of source-localized MEG activity [31]. Jonmohamadi et al. showed through simulations that source-based independent component analysis (ICA) reduced source localization errors as compared to source-localized sensor ICs [32]. Source localization was combined with EMD in a recent study [33], where EMD was used to recover similar event-related modes across electrodes which were then source-localized to their neuronal origin.

Spatial and temporal signal decomposition is relevant for effective brain stimulation, such as TMS. In TMS, a magnetic coil creates a time-varying electromagnetic field which induces an electrical current in the underlying area [34]. The impact on the brain is a function of where TMS is administered, the state of the given brain area, and the details of the stimulation [35]. These factors produce large interindividual and intraindividual differences in the responsiveness and therefore also in the therapeutic gain of brain stimulation [36, 37]. Stimulating the brain while in an advantageous state, i.e., when stimulation is most effective, is therefore important for treatment outcome. Focusing on the neurophysiological factors, the phase of the neuronal oscillations at and before stimulus onset has been shown to be a determinant for effective brain stimulation [8–11]. The motor-evoked potential (MEP) has been suggested as an indirect measure of motor cortex excitability, i.e., a large MEP indicates that the brain was stimulated while being in an advantageous state, and vice versa. However, for a full explanation of the MEP response, the excitability of the whole cortical and spinal system must be considered [38, 39]. A TMS-elicited MEP is created by placing the TMS coil above the M1 area which induces electrical currents in the tissue below the coil. This in turn causes a contraction of the contralateral muscle, with respect to the hemisphere being stimulated. Surface electromyography electrodes are placed on the target muscle, and they quantify the MEP [38]. Berger et al. found a correlation between the MEP response and the phase of the prestimuli neuronal oscillations within the alpha, slow and fast beta, and slow and fast gamma bands [8], and Keil et al. reported a correlation between MEP magnitude and phase of the EEG oscillations within the beta band [10].

In this work, we extend on the study of source-based signal decomposition by investigating the potential benefits obtained from performing frequency analysis on the cortical sources. More specifically, we propose to use source localization to recover the brain activation and then submit the recovered sources to frequency analysis using the data-adaptive method, EMD. It is hypothesized that performing EMD on the cortical sources as an alternative to electrode-level analysis will provide a more powerful analysis, as biological noise signals are potentially unmixed from the signal of interest. First, this hypothesis is tested through simulations in which we mimic realistic brain activation scenarios. In addition to comparing the accuracy of EMD performed on electrode and cortical-level signal, we perform EMD of each electrode and then perform source localization of the recovered modes across electrodes, as suggested by Al-Subari et al. [33]. Next, we analyze EEG data recorded while TMS was administered over the primary motor cortex. Similarly, to the study performed by Berger et al., we investigate the correlation between the magnitude of the MEP and the phase of the localized sources' temporal dynamics in prespecified frequency bands in a pre-TMS time window. However, we use EMD to decompose the signal instead of defining a wavelet basis [8], and we furthermore compare our results to performing electrode-level frequency analysis.

## 2. Materials and Methods

### 2.1. Notation

Vectors/matrices are denoted using bold lowercase/uppercase symbols, respectively. Scalars are in italics. Time series variables, such as **x**, are emphasized by the notation *x*(*t*), where *x*(*t*) is an element in the vector **x**. Subscripts demonstrate either indices (aside from time indices) or clarify affiliation of the variable. This should be clear from the context and through the definition of the variable.

### 2.2. Empirical Mode Decomposition

EMD decomposes the signal **x** ∈ *ℝ* ^*T*^ into a set of *W* zero-mean, narrow-band, amplitude, and frequency-modulated components, **c**_*w*_ ∈ *ℝ* ^*T*^, termed intrinsic mode functions (IMFs) [12–14]. The instantaneous amplitude, frequency, and phase of these components can be estimated via the Hilbert transform:

(1) $$\begin{array}{c} x\left(t\right)=\overset{W}{\underset{w=1}{\sum }}c_{w}\left(t\right)=\overset{W}{\underset{w=1}{\sum }}a_{w}\left(t\right)\psi_{w}\left(t\right)+r\left(t\right), \end{array}$$

where *ψ*_*w*_(*t*) is the oscillation of component *w* at time point *t* which has amplitude *a*_*w*_(*t*). Finally, *r*(*t*) contains the residual nonoscillating trend.

**Figure 1:**
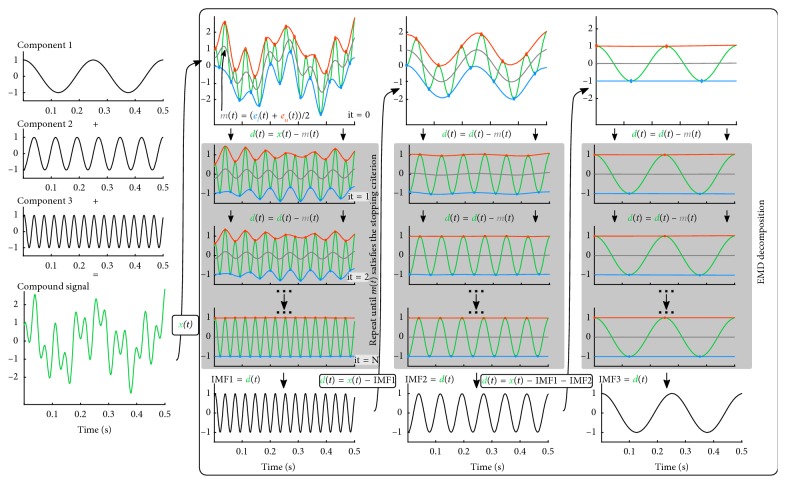
EMD sifting process. The components of the true signal and the compound signal (*x*(*t*)) are seen in the first column. In the second column, sifting is initiated (iteration 0) by finding the local peaks of the signal *x*(*t*) followed by interpolation of all minima (blue) and maxima (red), respectively. In iteration 1, the mean of the lower and upper envelope (gray, *m*(*t*)) is subtracted from the original signal, forming *d*(*t*). The local peaks are estimated for *d*(*t*), and as before, these are used to generate the lower and upper envelope. The process is repeated until the IMF conditions are fulfilled. Additional IMFs are extracted by sifting the residual of the original signal and the already obtained IMF(s).

An iterative process called *sifting* forms the IMFs such that each IMF fulfills two conditions. (1) The sample-wise mean of the upper and lower IMF envelopes is zero. (2) The number of zero crossings and extrema of the IMF does not differ by more than 1. These conditions ensure that the Hilbert transform does not yield negative values for the instantaneous frequencies. The sifting process in which the IMFs are recovered is visualized in Figure 1. In the example, the compound signal consists of three components having oscillations with frequencies 4, 13, and 18 Hz, respectively. Although this example contains only stationary components, it is important to note that EMD is also applicable to nonstationary signal components.

EMD is initiated (it = 0 in Figure 1) by finding the local peaks of the signal **x**, and from these, the lower **e**_*l*_ ∈ *ℝ* ^*T*^ and upper **e**_*u*_ ∈ *ℝ* ^*T*^ envelopes of the signal are created by cubic spline interpolation between the local minima and maxima, respectively. From the envelopes, the local mean at time instant *t*=1,…, *T* is approximated:

(2) $$\begin{array}{c} m\left(t\right)=\frac{e_{u}\left(t\right)+e_{l}\left(t\right)}{2}. \end{array}$$

In the next step (it = 1 in Figure 1), the local mean is subtracted from the original signal. The process of finding local peaks and forming envelopes is now repeated for the resulting signal until the two IMF conditions are fulfilled. Additional IMFs are extracted by repeating the sifting process on the residual of the original signal and the already obtained IMF(s). For further information on EMD, we refer to [12], where Huang et al. meticulously describe the method as well as its strength over Fourier and wavelet analysis. The benefits of EMD are furthermore demonstrated on several real-life examples.

#### 2.2.1. Multivariate EMD

The so-called multivariate EMD (MEMD) simultaneously recovers IMFs from multichannel data and ensures that (frequency) modes of the recovered IMFs are aligned across channels and IMF indices [14, 40]. As the extrema are usually not well defined for multivariate signals, Rehman and Mandic suggest generating *K* separate univariate signals by projecting the *n*-variate signal **x**(*t*) along *K* direction vectors in the *n*-dimensional space. For each projection of the signal, the minima (maxima) are located in time and the signal is then interpolated at these time points along each dimension resulting in approximates of the lower (upper) envelopes, **e**_*l*,*k*_(*t*) ∈ *ℝ* ^*n*^ (**e**_*u*,*k*_(*t*) ∈ *ℝ* ^*n*^). The local means **m**(*t*) ∈ *ℝ* ^*n*^ can be estimated as the average over the lower and upper envelopes of the *K* projections:

(3) $$\begin{array}{c} m\left(t\right)=\frac{1}{2K}\underset{k}{\sum }e_{u,k}\left(t\right)+e_{l,k}\left(t\right). \end{array}$$

The sifting process proceeds as for EMD, where **d**(*t*)=**x**(*t*) − **m**(*t*) in iteration 1 (Figure 1) and by **d**(*t*)=**d**(*t*) − **m**(*t*) in the following iterations. The lack of well-defined extrema complicates the IMF condition entailing counting extrema and is therefore not imposed.

#### 2.2.2. Noise-Assisted MEMD

*Mode mixing* is a known phenomenon occurring in both EMD and MEMD, wherein either several oscillatory components are contained in one IMF or one component is split between several IMFs [14]. To alleviate this problem, the so-called noise-assisted version of MEMD (NA-MEMD) was developed [41]. NA-MEMD exploits the effect EMD has when applied to a signal containing Gaussian white noise, wherein it acts as a quasi-dyadic filterbank on the recovered IMFs. By definition, white noise has a broad range of frequencies and this in turn ensures that the reconstructed IMFs collectively cover a wide range of frequency sub-bands [41]. To ensure that the IMFs are better aligned with the sub-bands recovered from the noise channels, Rehman and Mandic suggest adding a number of noise channels and performing MEMD on the composite signal [41]. The IMFs belonging to the noise channels are discarded in the following analysis.

Small leakages from the noise channels to the IMFs of the input signal can, however, occur. In these cases, Rehman and Mandic suggest repeating the NA-MEMD process for several realizations of noise and then averaging the IMFs belonging to the input signal across realizations. Finally, it is noted that NA-MEMD is only suitable when the quasidyadic filter bank is appropriate for the input signal, i.e., when the signal components approximately belong to the filterbank.

We use the toolbox created by Rehman and Mandic available at http://www.commsp.ee.ic.ac.uk/∼mandic/research/emd.htm (see the publication “Multivariate Empirical Mode Decomposition”) as the foundation of our NA-MEMD analysis. The implementation of NA-MEMD can be found at: https://github.com/STherese/NA-MEMD-for-EEG.git.

### 2.3. Source Localization

The mapping of cortical sources to scalp electrodes can be described by the linear forward problem:

(4) $$\begin{array}{c} y\left(t\right)=\mathrm{Ls}\left(t\right)+\epsilon \left(t\right). \end{array}$$

The forward model (or leadfield matrix), **L** ∈ *ℝ* ^*N*×*D*^, thus maps *D* sources to *N* electrodes in *T* time samples. The noise term, *ϵ*(*t*), is often modeled as being zero-mean and Gaussian-distributed [42]. A realistic forward model can be constructed by solving Poisson's equation in a physical model based on head anatomy as well as head-layer conductivities [3]. In an effort to improve construction of the forward model, studies have used the EEG data of the subject to further optimize the forward model [43–45]. However, in the following, we build the forward model based on subject anatomy and furthermore assume fixed orientations of the dipoles.

Solving the inverse problem of EEG is an ill-posed problem, as the locations of the cortical sources of EEG are in the order of thousands, while the number of sensors is, at most, in hundreds. Common simplifications to improve the uniqueness of the solution to the inverse problem include assuming smooth and focal sources [20, 23, 27]. These assumptions can be justified based on physiological knowledge of the brain. For instance, the neuronal generators of EEG are believed to be macrocolumns of cortical pyramidal neurons having coherent activity [19]. To provide a measurable signal at the scalp, a large number of neurons must be synchronously activated, often assumed to make up areas of order 5 × 5  mm. There are several methods that provide smooth source densities. Among these, we have chosen to work with a version of the well-known low-resolution electromagnetic tomography (LORETA) model. The specific algorithm is included in the freely available software package SPM12 [23, 42] for MATLAB (The MathWorks, Inc.).

In the LORETA version applied, source inference is approached by variational Bayes. By optimization of the free energy (a bound on the log evidence), relevant source components are weighed through their hyperparameters. The evidence of the model given the data is thus sought maximized by using the data to infer an appropriate source density. We refer to the Appendix section and references [23, 42] for further details on the specific source reconstruction procedure.

### 2.4. Pipeline

**Figure 2:**
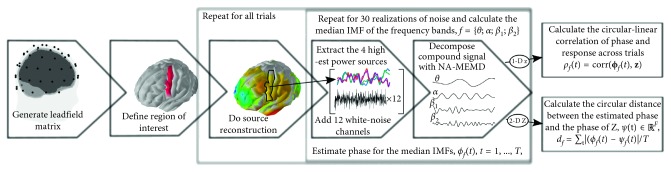
The pipeline used to perform NA-MEMD of the source-localized cortical activity in the ROI. Either 1 or 4 sources are extracted from the ROI. In the experimental data, the estimated phase is correlated with the 1-D (trials) MEP intensities (denoted **z**) using the circular-linear correlation. In the simulations where the true signal is available, it is compared directly to the estimated signal, i.e., through calculating circular distance between the phase of the true (*ψ* ∈ *ℝ* ^*F*×*T*^) and estimated signal (*ϕ* ∈ *ℝ* ^*F*×*T*^) in the frequency bands *f*=1,…, *F*.

The process used to decompose the signal spatially and temporally is summarized in Figure 2.

The leadfield matrix is generated in BrainStorm [46] by adapting the segmented head layers of the ICBM152 template to fit the size of the subject's head layers which are inferred from a subject MRI. From the adapted template cortex, inner skull, outer skull, and head together with the subject's coregistered EEG electrodes, an OpenMEEG BEM leadfield matrix [47] is constructed. Dependence in the choice of EEG reference montage is reduced by using the reference electrode standardization technique (REST) [48, 49]. The EEG data are thus analyzed in the REST domain, and the leadfield matrix is kept “reference-free.”

The defined region of interest (ROI) is based on prior knowledge in relation to the study paradigm. We thus define the ROI to contain the vertices belonging to the precentral gyrus, as it includes the anatomical location of the “motor hand area.” We use the Desikan–Killiany Atlas included in the ICBM152 segmentation from BrainStorm to define the left precentral gyrus. From the ROI, the temporal dynamics of either the one source (analyzed using EMD) or the four sources (analyzed using MEMD) having highest power are extracted and further analyzed. Using 12 noise channels, NA-MEMD is performed and the IMFs (belonging to the data channels) with highest power in the frequency bands: *θ*=[4,8]  Hz, *α*=[8,14]  Hz, *β*_1_=[14,22]  Hz, and *β*_2_=[22,30]  Hz are extracted. To minimize the effects of leakage of noise to the data channel(s), the NA-MEMD procedure is repeated 30 times with different realizations of white Gaussian noise. A median IMF is computed from the 30 extracted IMFs in each frequency band of interest such that one median IMF remains for each of the four bands. The whole procedure is repeated for each trial in the dataset. The extraction of trials is defined in the Simulated Data and Experimental Data sections.

We compare our results to a sensor-level analysis and thus perform NA-MEMD on the one (four) electrode(s) closest to the SOI. We furthermore compare our results to performing EMD before doing source localization and to applying bandpass filters to extract the components from the source-localized activity. For the latter, we use code from M. Cohen (http://mikexcohen.com/book/AnalyzingNeuralTimeSeriesData_MatlabCode.zip) (chapter 14) [50] to design FIR filters with transition widths of 20% and flexible filter order (depending on the frequency band). Before filter application, we employ a Hamming window. The cutoff frequencies are the same as used to extract the IMF components, i.e., corresponding to the *θ*, *α*, *β*_1_, and *β*_2_ boundaries.

### 2.5. Phase Correlation

In our analysis, we correlate the extracted phase with either a 1D or a 2D signal. We use the circular-linear correlation [51] when correlating the estimated phase across Nt trials for each time sample and frequency band, *ϕ*_*f*_(*t*) ∈ *ℝ* ^Nt^, with a 1D linear response signal, **z** (i.e., the MEP). The circular-linear correlation is given by

(5) $$\begin{array}{c} \rho_{f}\left(t\right)=\sqrt{\frac{r_{z,\cos\;\phi_{f}\left(t\right)}^{2}+r_{z,\sin\;\phi_{f}\left(t\right)}^{2}-2r_{z,\cos\;\phi_{f}\left(t\right)}r_{z,\sin\;\phi_{f}\left(t\right)}r_{\phi }}{1-r_{\phi }}}, \end{array}$$

where *r*_**a**,**b**_ is the Pearson correlation coefficient measuring the linear correlation of vectors **a** and **b** and *r*_*ϕ*_=*r*_cos *ϕ*_*f*_(*t*),sin *ϕ*_*f*_(*t*)_. The circular-linear correlation returns a value between 0 and 1. To account for multiple comparisons stemming from multiple time samples, we perform a cluster permutation test (see Section 2.6).

In the simulation study, we have ground truth and can therefore compute the difference between the phases of the estimated and true 2D signals directly. The phase difference is computed using the circular phase difference (see Figure 2 for details).

We use the freely available MATLAB toolbox CircStat [52] to compute both the circular-linear correlation and the circular phase difference.

### 2.6. Statistical Analysis

When testing for significant differences between how well the signals are reconstructed in the simulated data, we perform a two-sided *t*-test (alpha = 0.05) between each pair of methods. Evidence of higher performance is reported if a method is significantly better than all other methods.

In the analysis of potential correlations between the brain signal and an external response, we perform tests of significance for multiple time samples within subjects. To account for multiple comparisons, we use the so-called cluster permutation test [53]. This is a nonparametric method in which, in our case, neighboring time samples are combined in clusters. We form the clusters based on time samples that have a significantly higher correlation with the MEP response across trials, i.e., based on *ρ*_*f*_(*t*) in equation (5), than permutations of the data according to a one-sided *t*-test and an alpha value of 0.05 (uncorrected). The significance level controlling the individual time samples is a design parameter and hence does not affect the validity of the following cluster statistics. The cluster statistics can be calculated in several ways; here, we follow the suggestions in [53] and use the sum of *t*-values obtained for the time samples in the cluster. We run 1000 permutations, for which we calculate the maximum cluster statistic and compare this against the statistic calculated for the unpermuted observed data. If a cluster has a cluster statistic which is higher than that of 95% of the permuted data sets, we report a significant correlation between brain dynamics and behavioral response.

### 2.7. Simulated Data

**Figure 3:**
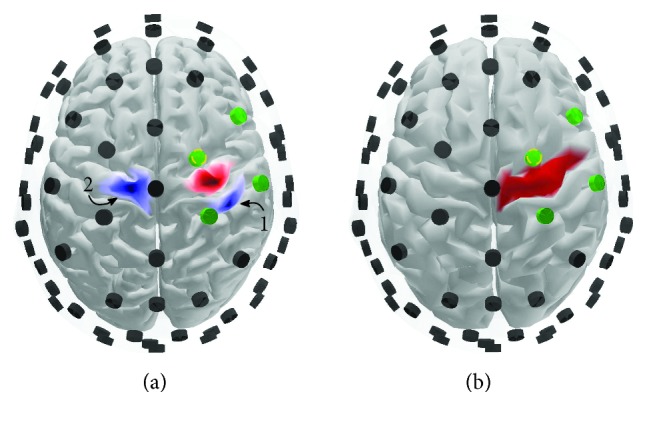
Source space of the synthetic data. (a) The cortex mesh used to generate the data (15,008 vertices) (the locations of the source of interest (red) and the distractor sources (blue)). (b) The cortex mesh (3,003 vertices) for building the forward model used for reconstruction. The red area depicts the upper part of the right precental gyrus and constitutes the region of interest. The electrodes are shown in black except for the four electrodes located closest to the SOI, and the nearest electrode is marked in yellow.

**Figure 4:**
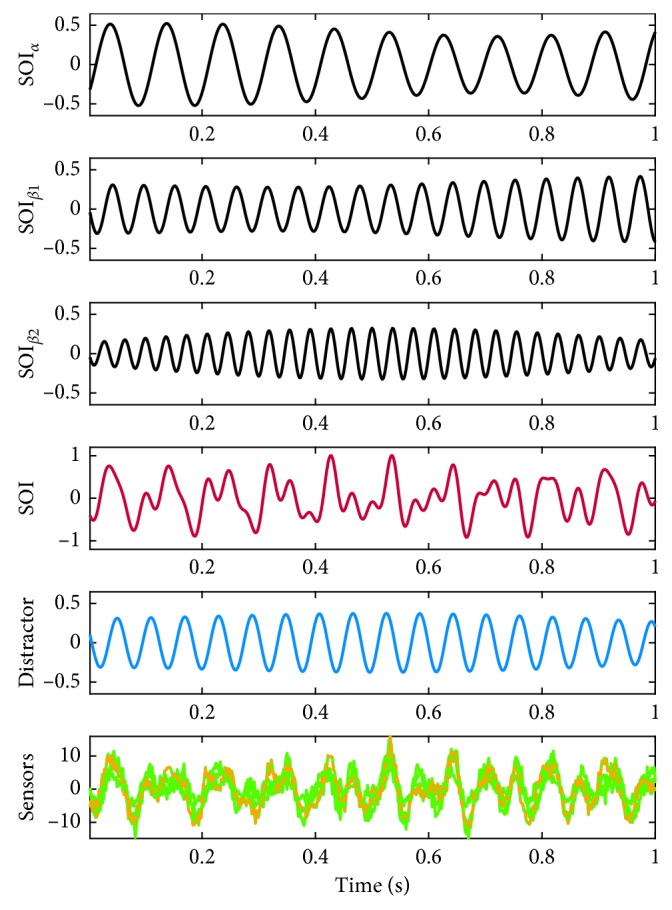
Example of the temporal dynamics of the synthetic data. Rows 1–3 depict the three components contained in the SOI, and their sum is seen in row 4. The temporal dynamics of the distractor is seen in blue in row 5. The projections of the SOI and the distractor to the four sensors located closest to the SOI are seen in the bottom row (with added noise). The signal of the nearest electrode is seen in dashed yellow/green.

We generated simulated data to test the performance of the noise-assisted MEMD when based on either the sensor or source data. The source space representation was constructed from the ICBM152 template anatomy available in Brainstorm [46], resulting in a source space consisting of 15,008 vertices restricted to the cortex surface. The activity of interest was placed in the right motor cortex (Figure 3(a)) and had the temporal dynamics seen in red in Figure 4. The temporal dynamics of the source of interest (**s**_SOI_) was thus a sum of three oscillations which varied ±0.5 Hz around the center frequencies 10.1, 18, and 27.5 Hz, i.e., modeling the SOI as having frequencies from the alpha and slow and fast beta ranges. The amplitude of the oscillations was designed to follow the 1/frequency power law. Each trial was 1 second long. One distractor source was included in each simulation. The distractor source oscillated ±0.5  Hz around a center frequency of 14 to 22 Hz. The possible locations of the distractor are visualized in blue in Figure 3(a), and an example of its temporal dynamics is shown as the blue trace in Figure 4.

In a second simulation study, we placed five distractors approximately 3, 5, 7, 9, and 11 cm from the SOI. The distances were jittered by up to 1 mm in order to obtain different placements across 100 repetitions. The SOI was generated as in the previously described simulations while the distractors each contained broadband oscillations in the 4 to 30 Hz range.

The SOI and distractor(s) were projected to 61 equidistant sensors in an M10 electrode layout (EASYCAP GmbH, Herrsching-Breitbrunn, Germany) using an OpenMEEG [47] boundary element forward model (**L**) generated in Brainstorm [46], and identically independently distributed (i.i.d.) noise (*ϵ*(*t*)) was added to give a specific signal-to-noise ratio:

(6) $$\begin{array}{c} \text{SNR}=20\cdot \log_{10}\frac{\text{std}\left(Ls_{\text{SOI}}\left(t\right)+Ls_{\text{distractor}}\left(t\right)\right)}{\text{std}\left(\epsilon \left(t\right)\right)}, \end{array}$$

where std denotes the standard deviation. In the simulations containing one distractor, the SNR was set to 0 dB, while when including five distractors the SNR was varied from −10 to 10 dB. 

For source reconstruction, we used an OpenMEEG forward model build from a cortex mesh with a resolution of 3,003 vertices (Figure 3). The resolution of the cortex was thus reduced for reconstruction in order to avoid the “inverse crime” [54]. We defined the region of interest in this mesh to be the upper part of the right precentral gyrus. This was done to mimic prior knowledge of a source located in the motor-cortex hand area.

Code for generating the synthetic data is available at https://github.com/STherese/NA-MEMD-for-EEG.git.

### 2.8. Experimental Data

The EEG signal of two subjects (both female and between 18 and 30 years) was recorded, while TMS pulses were administered over the right-hand motor area. Using a MagVenture PowerMag 100 Option TMS stimulator equipped with a figure-of-eight coil (Magventure, Denmark), 300 monophasic pulse stimuli were administered with an interstimuli interval of 8 ± 4  s. Maximum stimulator output was in the described setup 141 A/*µ*s. Prior to initiating the main experiment, the stimulator intensity was adjusted in order to reach a MEP intensity of approximately 1 mV using an adapted threshold-hunting algorithm based on the PEST procedure [55]. The MEP intensity was measured from the first dorsal interosseous muscle by electromyogram surface electrodes. Post hoc analysis revealed an average MEP of 0.5 mV for subject 1 (stimulation intensity: 75% of stimulator output) and 1.3 mV for subject 2 (stimulation intensity: 64% of stimulator output) (see Figures S4 and S5 for distributions of the MEP peak-to-peak amplitudes).

EEG was recorded from a 63 channel electrode cap (EasyCap, GmbH) suitable for TMS stimulation using an 80 channel NeurOne (Bittium Biosignals Ltd., Finland) with a sampling rate of 5,000 Hz. The data were downsampled to 1,000 Hz in the analysis. A subject-specific forward model was constructed from a T1-weighted MRI as previously described. The structural MRI scans were acquired on a Philips 3T Achieva MRI scanner (Philips Medical Systems, Best Netherlands) using a three-dimensional T1-weighted sagittal magnetization-prepared rapid acquisition gradient echo (MPRAGE) sequence (TR = 6 ms, TE = 2.70 ms; flip-angle = 8°, 0.85 mm isotropic voxel size, FOV = 245 × 245 × 208 mm).

Trials containing EEG of unusual high variance or trials with eyeblinks or prestimulus motor activity were manually removed, leaving 144 trials for subject 1. As seen in Figure S5, subject 2 had a relatively large number of trials with MEPs of high amplitude. Trials with MEP intensity above 1 mV were therefore removed, leaving 85 trials for subject 2. Subsequently, 25 trials having MEPs with amplitudes around the median MEP amplitude were removed, leaving in total 119 trials for subject 1 and 60 for subject 2. The median MEP amplitude trials were discarded in an attempt to focus further analysis on trials where motor cortex excitability was either relatively low or high. The EEG signal was epoched in the TMS prestimuli interval from −500  ms to −22  ms. All trials were then zero-padded to 2,479 samples and bandpass-filtered in the range [1,45]  Hz with a 5th order Butterworth filter (forward and backward direction). Zero-padding was added before (and removed after) bandpass filtering to reduce edge effects from filtering of the relatively short signal.

All subjects gave informed written consent prior to participating in the study, and the Health Research Ethics Committee of the Capital Region of Denmark approved the experiments. TMS was administered following TMS safety guidelines [7].

## 3. Results

### 3.1. Simulation Study

The extraction quality of the decomposed oscillations was quantified by their similarity to the simulated signal of interest by calculating the differences in phase and frequency, as well as the temporal correlation between the true SOI and the estimated signal.

In Table 1, we compare the extraction quality of NA-MEMD applied to the sensors against NA-MEMD on the sources of the ROI. The electrodes included in the sensor-level decomposition can be seen in Figure 3. “1 sensor” corresponds to NA-EMD of the electrode closest to the SOI (green and yellow dashed electrode in Figure 3), “4 sensors” additionally include the second, third, and fourth closest electrodes (green in Figure 3), and thus, NA-MEMD is performed here. Finally, “1 + sensors” indicates that NA-MEMD was performed on all four electrodes, but only the IMFs from the electrode closest to the SOI were analyzed. Thus, in the “1 + sensors” scenario, the three extra electrodes were merely used to support the retrieval of the IMFs belonging to the electrode nearest the SOI. Similarly, “1 source” is NA-EMD performed on the source having highest power in the ROI (Figure 3(b)), “4 sources” is NA-MEMD on the four sources having highest power in the ROI, and “1 + sources” uses the four strongest sources for NA-MEMD but only extracts IMFs from the strongest source.

In addition, we also tested NA-EMD applied to all sensors followed by source localization of the relevant IMFs (“EMD + SL”) and bandpass filtering of the source having maximum power in the ROI (“Bandpass”).

We see in Table 1 that the source-level decompositions improve on the “EMD + SL” and the sensor-level configurations for all averages. Especially in the scenario where the distractor is placed close to the SOI (distractor 1) is the “4 sensors” far outperformed. However, it seems that, if the distractor is further away from the SOI, and therefore also further away from the four electrodes used for decomposition, as is the case for distractor 2, the difference in performance becomes smaller. The bandpass filter method approaches the performance of the source level decompositions for both distractor locations with respect to deviation in phase and frequency.

Analyzing the results at an even more detailed level, Figures S1 and S2 reveal that the distractor source starts causing damage when its oscillation has a frequency approaching that of the oscillations of the SOI source components. The source-level decompositions are most robust to this effect and are more stable across distractor frequencies while the “EMD + SL,” “Bandpass,” and “4 sensors” are especially affected by this phenomenon. Between the three sensor-level decomposition, “1 sensor” achieves highest performance. Furthermore in the retrieval of the *β*_2_ component, “1 sensor” takes overall preference for the two single distractor placements (Figures S1 and S2) until the distractor frequency is ∼21 Hz.

Across SNRs (Figure S3) source-level decompositions are generally better or on par with the sensor-level decompositions, “Bandpass,” and “EMD + SL” solutions. For very low SNRs “Bandpass,” “1 sensor,” and “1 + sensor” obtains best performance. At ∼−5 dB, preference is shifted to the source-level decompositions for most performance measures.

The computational complexity of the methods was highly influenced by the number of times the MEMD was run. On a laptop (16 GB RAM, 2.6 GHz CPU), one realization with *K*=16 projection directions and 12 noise channels took approximately 1 second. As 30 NA-MEMD realizations were run, the computation time for each of the sensor configurations was approximately 30 s. Source localization took approximately 1 s, and the computation time of the source + EMD configurations were therefore only a bit longer. The “EMD + SL” performs EMD on all 61 electrodes, and its computation time therefore reached approximately 30 min. The “Bandpass” method was the fastest taking approximately 25 ms (excl. source localization).

### 3.2. Experimental Study

**Figure 5:**
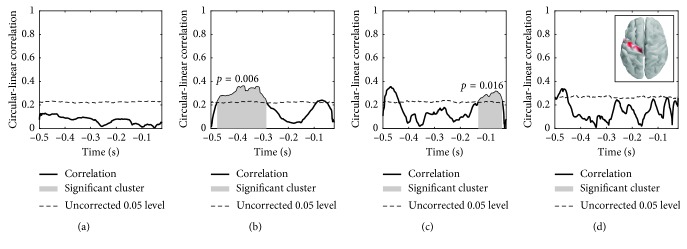
Subject 1: significant clusters from a temporal cluster permutation test, source-level. The circular-linear correlation across trials was calculated between the MEP response and the phase of the extracted components in four frequency bands. The components were extracted from the source having highest power in the LPG. The locations of this source across trials are indicated in the inset. Light red: few trials having highest power here; dark red: most trials having highest power here. (a) *θ* (4–8 Hz). (b) *α* (8–14 Hz). (c) *β*_1_ (14–22 Hz). (d) *β*_2_ (22–30 Hz).

We performed cluster permutation tests to quantify significant correlations between the MEP response and the decomposed neuronal oscillations in four frequency bands of interest. The simulations showed that the “1 sensor” and “1 source” achieved highest performance within the sensor- and source-level decompositions, respectively. These configurations were therefore tested on the experimental data. The results for subject 1 in the “1 source” setup, which entailed NA-EMD of the source of highest power in the left precental gyrus (LPG), are seen in Figure 5. Significant temporal clusters were found within the *α* and *β*_1_ bands in the time intervals (with respect to TMS onset) *τ*_*α*_=[−0.476, −0.285]  s and *τ*_*β*_1__=[−0.130, −0.038]  s, respectively.

**Figure 6:**
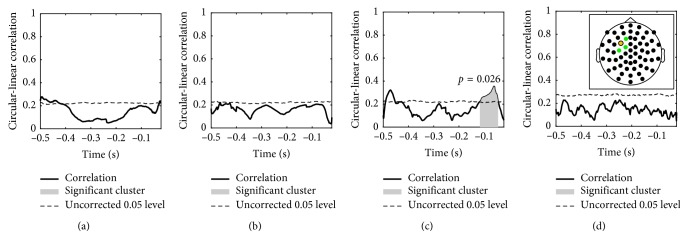
Subject 1: significant cluster from a temporal cluster permutation test, sensor level. The circular-linear correlation across trials was calculated between the MEP response and the phase of the extracted components in four frequency bands. The components were extracted from the sensor closest to the TMS entry point, marked yellow in the inset. (a) *θ* (4–8 Hz). (b) *α* (8–14 Hz). (c) *β*_1_ (14–22 Hz). (d) *β*_2_ (22–30 Hz).

**Figure 7:**
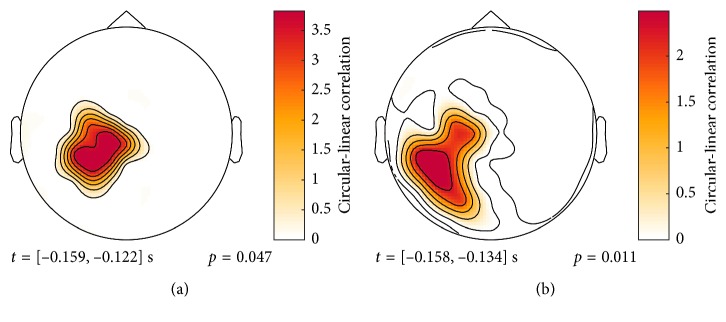
Subject 1: significant clusters of a 2D (sensor, time) cluster permutation test. The temporally summed, squared circular-linear correlation calculated between all sensors and the MEP response across trials. (a) *β*_1_ (14–22 Hz). (b) *β*_2_ (22–30 Hz).

The cluster permutation test for subject 1 also revealed a significant cluster for the *β*_1_ band sensor-level IMF (Figure 6). The cluster was placed in a similar time interval, *τ*_*β*_1__=[−0.117, −0.045]  s, as in the source-level analysis. The 2D cluster permutation across electrodes and time samples also yielded a significant cluster in the *β*_1_ band and also in a similar time interval (Figure 7). A significant cluster was also found in the *β*_2_ band. Both clusters were close to the TMS entry point, however, especially prominent for the *β*_2_ component, extending posteriorly and laterally. The bandpass filter approach was also tested on the localized source of highest power. Significant clusters were found in the *α* and *β* bands, both close to TMS onset (Figure S10).

**Figure 8:**
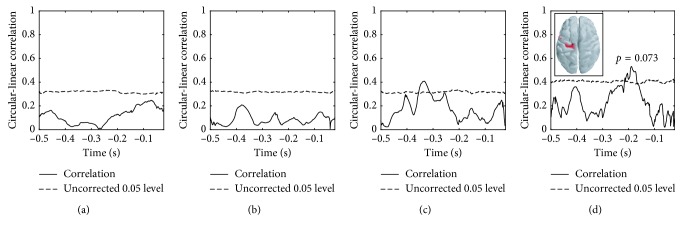
Subject 2: absence of significant cluster from a temporal cluster permutation test, source level. The circular-linear correlation across trials was calculated between the MEP response and the phase of the extracted components in four frequency bands. The components were extracted from the source having highest power in the LPG. The locations of this source across trials are indicated in the inset. Light red: few trials having highest power here; dark red: most trials having highest power here. (a) *θ* (4–8 Hz). (b) *α* (8–14 Hz). (c) *β*_1_ (14–22 Hz). (d) *β*_2_ (22–30 Hz).

**Figure 9:**
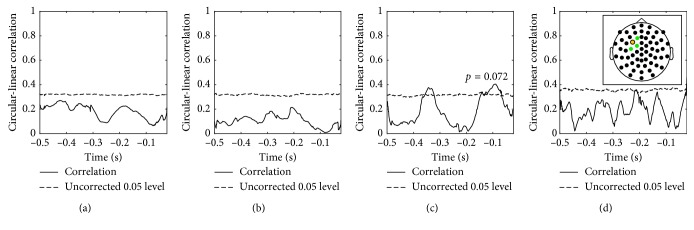
Subject 2: absence of significant clusters from a temporal cluster permutation test, sensor level. The circular-linear correlation across trials was calculated between the MEP response and the phase of the extracted components in four frequency bands. The components were extracted from the sensor closest to the TMS entry point, marked yellow in the inset. (a) *θ* (4–8 Hz). (b) *α* (8–14 Hz). (c) *β*_1_ (14–22 Hz). (d) *β*_2_ (22–30 Hz).

The corresponding results for subject 2 are seen in Figures 8 and 9. The sensor- and source-level 1D cluster permutation test and the 2D (sensor, time) analysis (not shown) did not provide any significant clusters.

Subject 2 had a relatively low number of accepted trials, and the analysis was therefore repeated with the median MEP intensity trials included. For the source-level analysis, this resulted in a significant correlation (*p*=0.034) between the subject's MEP response and the extracted *β*_2_ IMF components in the time interval [−0.217, −0.160]  s (Figure S8). The bandpass filter approach was also tested but did not recover any significant clusters.

## 4. Discussion

Neuronal activity can be characterized by its spatial and temporal properties. Source localization of EEG or MEG is becoming a viable way to obtain these properties. Working with electrical activity (or derivations thereof), as opposed to, e.g., the hemodynamic response, has the benefit of permitting temporal analysis of the fast-changing signal and allows decomposition into components having specific modes with respect to frequency, phase, and amplitude (see, e.g., [56–58]). Separating the signal into several components can strengthen subsequent analysis and assist in determining the connection between neuronal activation and behavioral responses.

In their seminal work on EMD, Huang et al. detailed and demonstrated its benefits compared to classical alternatives for frequency analysis, e.g., Fourier and wavelet analysis [12]. Mandic et al. further showed that EMD provides a clearer separation in time and frequency compared to wavelets and short-time Fourier transform spectrograms [14]. The advantages of NA-MEMD have been demonstrated by Alegre-Cortés et al. in connection with analysis of the oscillations of neuronal populations [59]. More specifically, they showed that the time-frequency resolution was increased and that more details concerning the oscillations could be obtained compared to linear frequency analysis approaches.

In the presented simulations, we tested whether (multivariate) empirical mode decomposition was capable of retrieving components of interest from a signal mixed with white noise and one or five distractor oscillations. In the single distractor design, the source of the distractor oscillation was either placed quite close to the source of interest (SOI) or at some distance, i.e., where it could or where it could not be expected to be picked up by the electrodes closest to the SOI. The distractor oscillated with a frequency and amplitude similar to the components of interest. In the five distractor setup, the distractors were placed both close and far from the SOI (3 to 11 cm) and they each had broadband oscillations (4 to 30 Hz).

The source-based decompositions achieved in general highest performances, and the extracted components from the NA-EMD on the source of highest power were most similar to the planted activity in most experiments. However, NA-EMD based on the closest SOI sensor achieved better retrieval of the *β*_2_ component when the distractor frequencies were below 21 Hz in the single distractor simulations. Looking into this phenomenon, we discovered that the electrode-level signal had a lower maximum frequency than the source-level signal. This in turn can be explained by the projection of sources to sensors through the scull which acts as a (here beneficial) lowpass filter. However, when the distractor frequency was above 21 Hz and thus approached the *β*_2_ component frequency of 27.5 Hz, decomposition of the highest power source took preference and undermined this effect. We furthermore note that the component specific SNR for the *β*_2_ oscillation is lower as the amplitude of the SOI oscillations are scaled according to frequency content while the noise is not generated to follow the power law. And, we do see in general that the sensor-level decomposition outperforms source-level decomposition when the SNR is very low (<−10 dB).

Focusing on the sensor-level performances, we see that when the distractor is close in placement and frequency to the SOI, it is clearly better only to use the nearest electrode, or at most only use additional electrodes to support the mode decomposition (i.e., the “1 + sensors” configuration). This indicates the importance of knowing which electrode is closest to the study-specific SOI. In many situations, it might be easier to define an anatomical region of interest rather than locating the most important electrode, and source-based decomposition is therefore a natural advancement. Here, we necessarily assume that the region of interest do not also include noise sources with similar but distinct oscillations.

Overall, in the single-distractor simulations, we observe that mode retrieval is most successful when the distractor oscillates with a different frequency than the SOI. The EMD and most other signal decompositions methods will naturally struggle to separate signals with similar properties. Furthermore, the chosen inverse solver, LORETA, is inherently challenged when subjected to source densities containing adjacent activations. Even so, we see that LORETA does provide beneficial unmixing of two similar sources. However, although most solvers will struggle in these scenarios, it would be interesting to expand the investigation to other solvers which do not enforce spatial smoothness in the source localization procedure, e.g., dipole fitting or beamformers.

Reversing the order in which source localization and EMD was implemented, i.e., similar to the technique presented in [33], was in most cases not better than the suggested procedure. This is likely due to the problem of matching the IMFs across all electrodes which is difficult when working with spontaneous EEG activity. Using EMD to find relevant signatures which are then source-reconstructed is more suitable when working with ERPs and would probably also benefit from a manual pairing of the IMFs. This was also originally proposed by Al-Subari et al. in [33] where they use EMD to find signatures in the EEG data related to their paradigm and then apply source reconstruction to locate their origins.

Employing simple bandpass filters to extract relevant components worked quite well when the SOI components were easily distinguishable from those of the distractors. However, the method is not capable of disentangling the noise components when they interfere with the components of the SOI.

The preliminary analysis of experimental data indicated that performing NA-MEMD on source-localized EEG increased the sensitivity of recovering correlations between neuronal oscillations and a behavioral response. The source- and sensor-level decompositions generally showed similar trends in the correlation values across time and frequency bands although decomposition of source-level data proved a stronger link between the behavioral response and the neuronal activity. Specifically, we recovered a higher correlation between the MEP response and the localized electrical activity than between the MEP and the sensor-level signal and furthermore recovered significant correlations in more frequency bands.

We controlled for multiple comparisons using cluster permutation tests on the temporal level, as well as on the spatial level in the 2D analysis. However, in the presented *p* values, we did not account for testing multiple frequency bands. This could, for example, have been handled with an expanded cluster permutation test, i.e., a 3D (space, time, frequency) analysis.

To our knowledge, comparisons of sensor- and source-level frequency analysis on this type of data have not been done previously. However, Keil et al. investigated the link between MEP size and phase of the EEG signal and found a significant correlation of 0.2 within the beta band oscillations [10]. Furthermore, in a group study, Berger et al. investigated the analogous source-level correlation and found significant correlations in the alpha band as well as in the slow and fast beta and gamma bands, with a maximum correlation of 0.15 [8]. Keil et al. used a simple bandpass filter of the EEG signal and Berger et al. performed wavelet decomposition on source-localized LPG activity. The higher correlation values (around 0.4) obtained in our experiment could be due to several factors such as a modified pipeline, the correlation between phase and MEP is subject-dependent with respect to frequency and timing, or simply because a different number of trials were included in our study. In line with the general observation of large intersubject variability in electrophysiology [36, 60], we find differences in our two subjects, the statistical tests show significant effects in both subjects, but responses are different. A future study including more subjects and trials is necessary to validate the preliminary results presented here. The necessity of removing the median MEP intensity trials should also here be investigated.

It seems counterintuitive that some of the significant temporal clusters are located far (up to 400 ms) from TMS onset. One should think that the instantaneous phase would become more informative when approaching the stimulation onset. When this is not the case, it can be explained by several factors. Firstly, noise-reducing filters produce edge effects, and the EMD, together with the phase estimation, is generally less accurate at the borders of the time window, explaining why there could be missing correlations in these areas. Second, the EEG signal is in general highly affected by noise which will affect the accuracy of the phase estimation. That is, the phase of the signal of interest is most accurately recovered if the signal's amplitude is high compared to the noise level. As the noise level is affected by many noise sources, biological and nonbiological, it will naturally vary across time causing the SNR to change and thus also affect the accuracy of the signal decomposition. Especially, the slower oscillations which are more coherent across time, e.g., in the alpha range, might therefore be correlated in earlier time windows and not in later. Increasing the number of trials should reduce this effect. Alternatively, a successful spatial unmixing of the signal should increase the SNR, which we also do see a trend towards in our analysis as more significant clusters of correlation are recovered when having applied source localization. Thirdly, Berger et al. [8] also reported significant correlations between the MEP size and the phase of brain oscillations in early time windows. They suggest that this could be caused by higher order inputs to the area which affect brain excitability at later stages.

In summary, the preliminary analysis of experimental data demonstrates a link between the magnitude of the MEP response and the decomposed neuronal oscillations on a single-subject level. We have demonstrated that this link can be recovered by empirical mode decomposition of localized electrical activity. Furthermore, both the results obtained in the simulation study and the experimental data lead us to conclude that source localization indeed provides an advantageous unmixing of the electrical signal captured by the EEG sensors. Thus, source localization provides more detailed information, which is beneficial for subsequent time-frequency analysis.

## Figures and Tables

**Table 1:** Performance on synthetic data (average deviation between estimated and actual source components). Performances are averaged across the three SOI components and the 100 repetitions. The simulations containing distractors 1 and 2 are additionally averaged across the distractor frequency, while the simulations containing five distractors are averaged across SNR levels.

	Deviation in phase (rad)	Deviation in frequency (Hz)	Temporal correlation
Distractor 1			
1 sensor	0.175	0.205	0.955
4 sensors	0.559	0.901	0.752
1 + sensors	0.209	0.221	0.932
1 source	**0.146** ^*∗*^	**0.173**	**0.972** ^*∗*^
4 sources	0.156	0.177	0.967
1 + sources	0.154	0.177	0.968
Bandpass	0.164	0.178	0.920
EMD + SL	0.484	0.720	0.799

Distractor 2			
1 sensor	0.208	0.276	0.939
4 sensors	0.218	0.231	0.932
1 + sensors	0.217	0.227	0.933
1 source	**0.164** ^*∗*^	**0.182**	**0.962** ^*∗*^
4 sources	0.193	0.198	0.947
1 + sources	0.173	**0.182**	0.957
Bandpass	0.211	0.204	0.900
EMD + SL	0.491	0.670	0.786

5 distractors			
1 sensor	0.432	0.474	0.830
4 sensors	0.570	0.525	0.739
1 + sensors	0.439	0.404	0.816
1 source	**0.390**	0.433	**0.850**
4 sources	0.391	0.352	0.844
1 + sources	0.396	**0.347**	0.842
Bandpass	0.452	0.480	0.791
EMD + SL	0.509	0.804	0.776

Significant improvements (^*∗*^ *p* < 0.001) are indicated. Applying a paired *t*-test did not change the significance pattern. Each of the source-level decompositions is significantly better than the sensor-level decompositions and the EMD + SL.

## Data Availability

Code for generating the synthetic data and scripts for computing NA-MEMD are available at https://github.com/STherese/NA-MEMD-for-EEG.git. Reduced EEG data for the two subjects are included in Supplementary Materials.

## Appendix

### The Applied Source Localization Approach

For source localization of the EEG generators, we use a variational Bayes version of LORETA as implemented in SPM12 [23, 42]. For completeness, we describe the source density estimation procedure, also detailed in [23, 42].

The forward problem, as presented in equation (4), describes the mapping of cortical sources (**S** ∈ *ℝ* ^*D*×*T*^) to the EEG sensors (**Y** ∈ *ℝ* ^*N*×*T*^) through the forward model (**L** ∈ *ℝ* ^*N*×*D*^). In the adapted LORETA version, the generalized solution to the inverse problem under assumptions of zero mean Gaussian noise with covariance **Q**_*ϵ*_ ∈ *ℝ* ^*N*×*N*^ and zero mean Gaussian sources with covariance **Q** ∈ *ℝ* ^*D*×*D*^ is formulated as [42]

(A.1) $$\begin{array}{c} S=QL^{T}{\left(Q_{\epsilon }+LQL^{T}\right)}^{-1}Y. \end{array}$$

The inference procedure as described by Friston et al. starts by reducing the input data using spatial and temporal projectors. Then, the model parameters, i.e., the sources, are eliminated from the model, such that they are explained by their hyperparameters. This in turn facilitates a reduced version of evidence maximization (EM). First, the so-called M-step is iterated until convergence in which the free energy is optimized over the hyperparameters. Then, one “E-step” is performed which provides the parameters.

The LORETA-like spatial prior covariance of source activity takes the form, **Q**=*h*_*I*_ **I**+*h*_*G*_ **G**, where **I** is the identity matrix of size *N* × *N* modeling independent equally weighted sources. And, **G** models coherent sources:

(A.2) $$\begin{array}{c} G=\overset{8}{\underset{i=0}{\sum }}\frac{\sigma^{i}}{i!}A^{i}, \end{array}$$

where **A** is the *N* × *N* adjacency matrix [23] and *σ*=[0,1] controls the smoothing degree, in the algorithm set to 0.6. The hyperparameters *h*_*I*_ and *h*_*G*_ are estimated using the EM procedure explained above, which thus determines the weighing of the “LORETA” and minimum norm priors. The hyperparameter of the noise covariance, which acts as a regularization parameter, is estimated simultaneously with *h*_*I*_ and *h*_*G*_. Once these have been estimated, they can be plugged into equation (A.1) through the covariance matrix, **Q**.

## Supplementary Materials

Supplementary Materials Data set for subject 1 (TMS EEG data sub1 subject.zip) includes the following: Trial: filtered and epoched EEG data, discard: which trials to discard, Gain: leadfield matrix, R: REST transformation matrix, SOI: sources in LPG, and chans LPG: electrodes nearest TMS stimuli. Data set for subject 2 (TMS EEG data sub2 subject.zip) includes the following: Trial: filtered and epoched EEG data, discard: which trials to discard, Gain: leadfield matrix, R: REST transformation matrix, SOI: sources in LPG, and chans LPG: electrodes nearest TMS stimuli.
