# Supplementary material for: Unmixing Oscillatory Brain Activity by EEG Source Localization and Empirical Mode Decomposition
Source: Comput Intell Neurosci. 2019 Mar 14;2019:5618303. doi: 10.1155/2019/5618303 (PMC6448348; doi:10.1155/2019/5618303)
Supplement: Supplementary Materials — Data set for subject 1 (TMS EEG data sub1 subject.zip) includes the following: Trial: filtered and epoched EEG data, discard: which trials to discard, Gain: leadfield matrix, R: REST transformation matrix, SOI: sources in LPG, and chans LPG: electrodes nearest TMS stimuli. Data set for subject 2 (TMS EEG data sub2 subject.zip) includes the following: Trial: filtered and epoched EEG data, discard: which trials to discard, Gain: leadfield matrix, R: REST transformation matrix, SOI: sources in LPG, and chans LPG: electrodes nearest TMS stimuli. [file 5618303.f1.zip › Supplmental_Figures.pdf]

Supplementary material to:
Unmixing oscillatory brain activity by EEG source localization and empirical mode decomposition

Additional simulation results

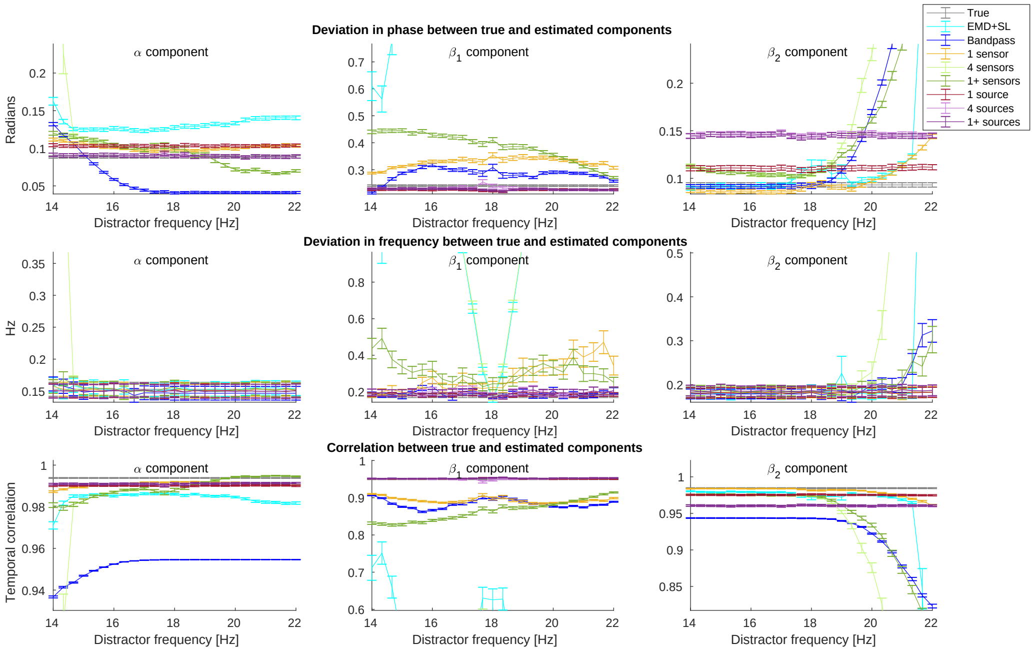

Figure S1: Simulation, distractor 1. NA-(M)EMD evaluated in sensor and source space for a distractor source in location 1, see Figure 3A. Rows 1 and 2 show deviation in phase and frequency, respectively, between estimated and true signal components. Row 3 shows the correlation between the estimated and true signal components. The line segments marked by "True" indicate the performance when decomposing the simulated SOI signal without noise. Note the axes for the β_1 component performance measures have been cropped to increase separability of the solutions, leading to "EMD+SL" and "4 sensors" being more or less cropped out.

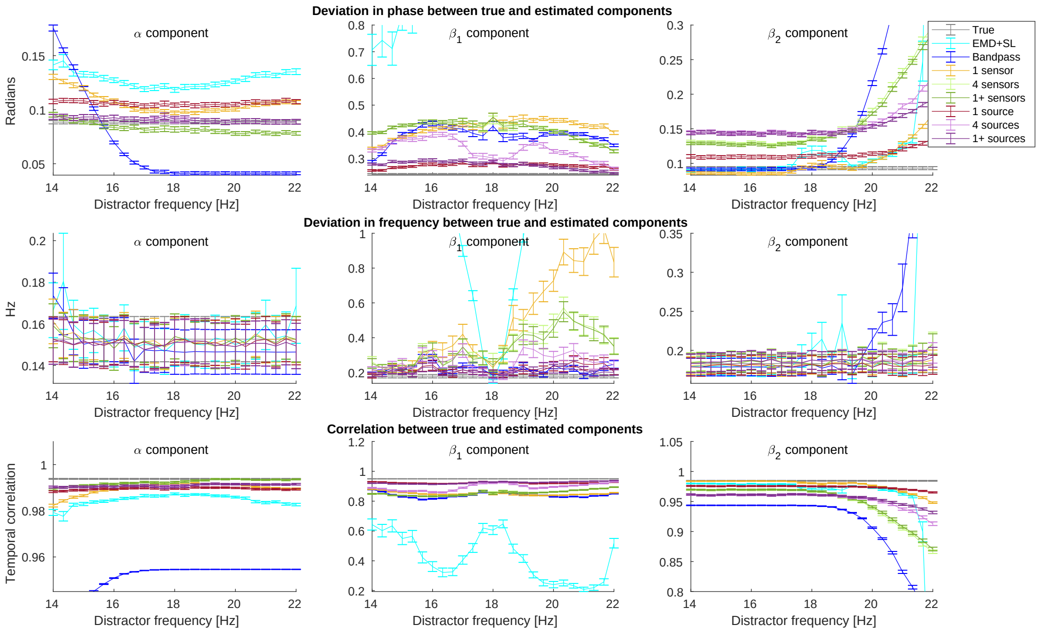

Figure S2: Simulation, distractor 2. NA-(M)EMD evaluated in sensor and source space for a distractor source in location 2, see Figure 3A. Rows 1 and 2 show deviation in phase and frequency, respectively, between estimated and true signal components. Row 3 shows the correlation between the estimated and true signal components. The line segments marked by "True" indicate the performance when decomposing the simulated SOI signal without noise.

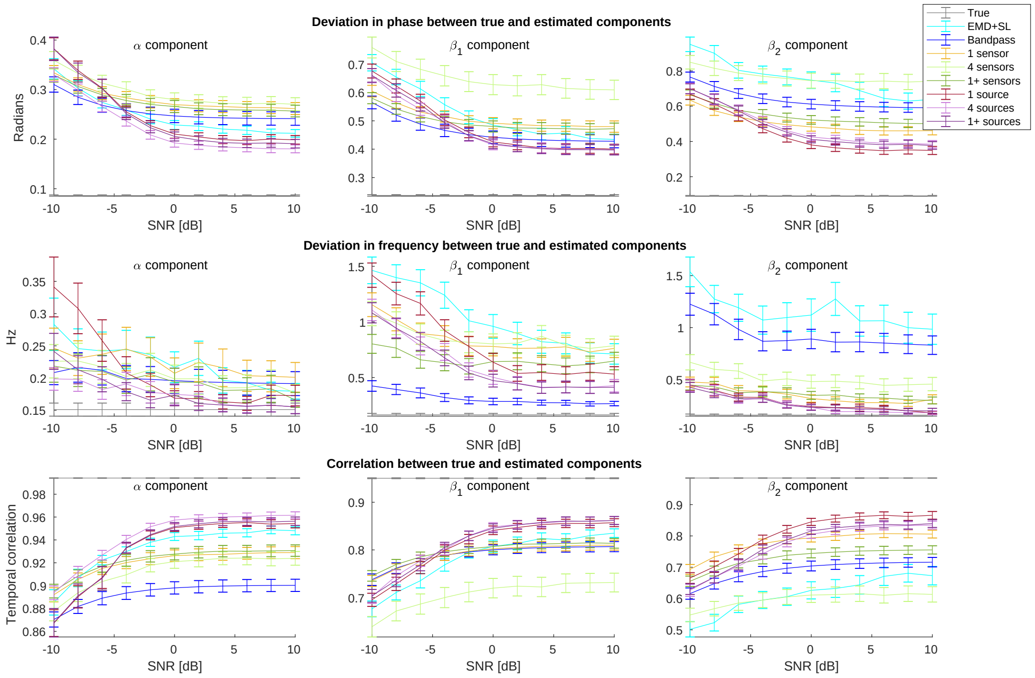

Figure S3: Simulation, five distractors. NA-(M)EMD evaluated in sensor and source space when including five distractor sources, placed 3, 5, 7, 9, and 11 cm from SOI. Rows 1 and 2 show deviation in phase and frequency, respectively, between estimated and true signal components. Row 3 shows the correlation between the estimated and true signal components, respectively. The line segments marked by "True" indicate the performance when decomposing the simulated SOI signal without noise.

Additional experimental results

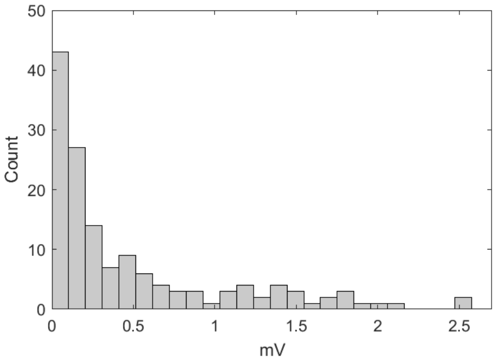

Figure S4: Histogram showing MEP magnitude for subject 1. MEP from all trials, except those marked as bad, are included.

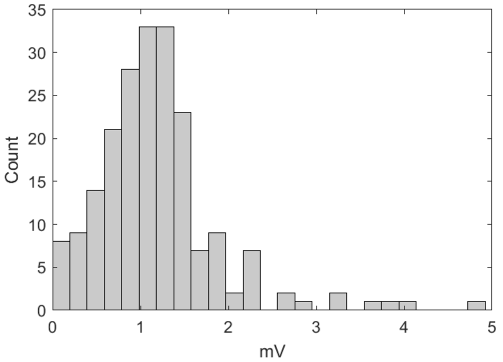

Figure S5: Histogram showing MEP magnitude for subject 2. MEP from all trials, except those marked as bad, are included.

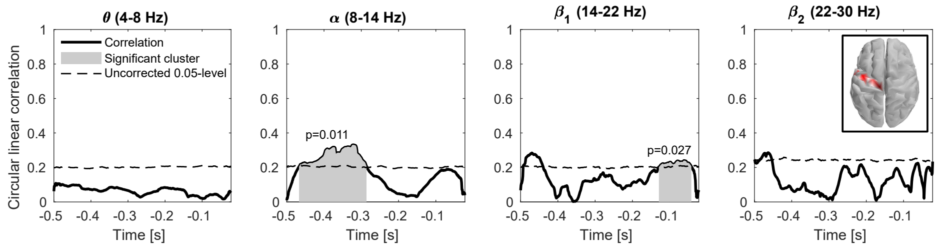

Figure S6: Subject 1: Significant clusters from a temporal cluster permutation test, source-level, including trials having median MEP intensity. The circular-linear correlation across trials was calculated between the MEP response and the phase of the extracted components in four frequency bands. The components were extracted from the source having highest power in the LPG. The locations of this source across trials are indicated in the inset. Light red: few trials having highest power here, dark red: most trials having highest power here.

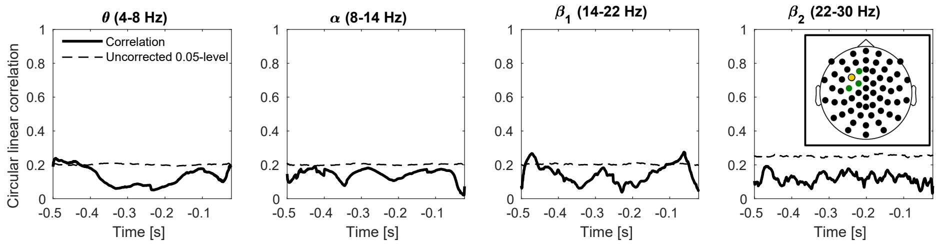

Figure S7: Subject 1: Significant clusters from a temporal cluster permutation test, sensor-level, including trials having median MEP intensity. The circular-linear correlation across trials was calculated between the MEP response and the phase of the extracted components in four frequency bands. The components were extracted from the sensor closest to the TMS entry point, marked yellow in the inset.

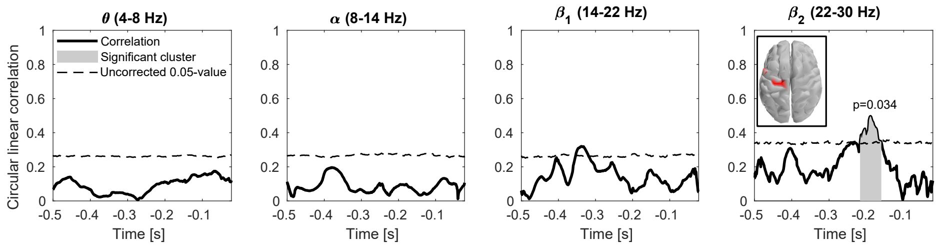

Figure S8: Subject 2: Significant cluster from a temporal cluster permutation test, source level, including trials having median MEP intensity. The circular-linear correlation across trials was calculated between the MEP response and the phase of the extracted components in four frequency bands. The components were extracted from the source having highest power in the LPG. The locations of this source across trials are indicated in the inset. Light red: few trials having highest power here, dark red: most trials having highest power here.

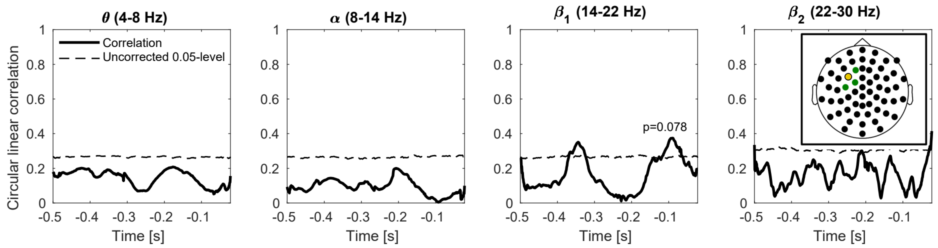

Figure S9: Subject 2: Absence of significant clusters from a temporal cluster permutation test, sensor level, including trials having median MEP intensity. The circular-linear correlation across trials was calculated between the MEP response and the phase of the extracted components in four frequency bands. The components were extracted from the sensor closest to the TMS entry point, marked yellow in the inset.

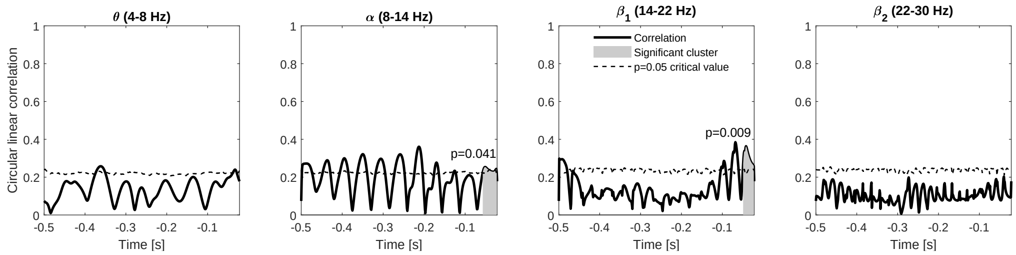

Figure S10: Subject 1 with bandpass filters: Significant clusters from a temporal cluster permutation test, source-level, excluding trials having median MEP intensity. The circular-linear correlation across trials was calculated between the MEP response and the phase of the extracted components in four frequency bands. The components were extracted from the source having highest power in the LPG.
